# Elevated δ^15^N Linked to Inhibited Nitrification Coupled to Ammonia Volatilization in Sediments of Shallow Alkaline‐Hypersaline Lakes

**DOI:** 10.1111/gbi.70018

**Published:** 2025-04-04

**Authors:** Christopher J. Tino, Eva E. Stüeken, Daniel D. Gregory, Timothy W. Lyons

**Affiliations:** ^1^ Department of Earth and Planetary Sciences University of California Riverside California USA; ^2^ Department of Earth, Energy, and Environment University of Calgary Calgary Alberta Canada; ^3^ School of Earth and Environmental Sciences University of St. Andrews St. Andrews UK; ^4^ Virtual Planetary Laboratory University of Washington Seattle Washington USA; ^5^ Department of Earth Sciences University of Toronto Toronto Ontario Canada

## Abstract

Alkaline lakes are among the most bioproductive aquatic ecosystems on Earth. The factors that ultimately limit productivity in these systems can vary, but nitrogen (N) cycling in particular has been shown to be adversely affected by high salinity, evidently due to the inhibition of nitrifying bacteria (i.e., those that convert ammonic species to nitrogen oxides). The coastal plain of Coorong National Park in South Australia, which hosts several alkaline lakes along 130 km of coastline, provides an ideal natural laboratory for examining how fine‐scale differences in the geochemistry of such environments can lead to broad variations in nitrogen cycling through time, as manifest in sedimentary δ^15^N. Moreover, the lakes provide a gradient of aqueous conditions that allows us to assess the effects of pH, salinity, and carbonate chemistry on the sedimentary record. We report a wide range of δ^15^N values (3.8‰–18.6‰) measured in the sediments (0–35 cm depth) of five lakes of the Coorong region. Additional data include major element abundances, carbonate δ^13^C and δ^18^O values, and the results of principal component analyses. Stable nitrogen isotopes and wt% sodium (Na) display positive correlation (*R* ^2^ = 0.59, *p* < 0.001) across all lake systems. Principal component analyses further support the notion that salinity has historically impacted nitrogen cycling. We propose that the inhibition of nitrification at elevated salinity may lead to the accumulation of ammonic species, which, when exposed to the water column, are prone to ammonia volatilization facilitated by intervals of elevated pH. This process is accompanied by a significant isotope fractionation effect, isotopically enriching the nitrogen that remains in the lake water. This nitrogen is eventually buried in the sediments, preserving a record of these combined processes. Analogous enrichments in the rock record may provide important constraints on past chemical conditions and their associated microbial ecologies. Specifically, ancient terrestrial aquatic systems with high δ^15^N values attributed to denitrification and thus oxygen deficiency may warrant re‐evaluation within the framework of this alternative. Constraints on pH as provided by elevated δ^15^N via ammonia volatilization may also inform critical aspects of closed‐basin paleoenvironments and their suitability for a de novo origin of life.

## 1 Introduction

Closed‐basin lacustrine environments provide a means of exploring the influence of various water chemistries and related physicochemical stressors (e.g., evapo‐concentration) on surface biogeochemistry. More specifically, they exhibit conditions that are not encountered in the modern ocean. Therefore, these settings can serve as natural testbeds for calibrating biogeochemical proxies under a wide range of conditions, including those which were perhaps prevalent on the early Earth (Cohen 2003). Closed‐basin lakes are also of astrobiological interest due to their capacity for wet‐dry cycles—thought by some to be a critical circumstance for the de novo emergence of life (Lahav et al. 1978; Campbell et al. 2019; Damer and Deamer 2020)—and they may inform the mineralogical products of desiccation events similar to those that occurred on Mars approximately 3–4 billion years ago (Stein et al. 2018; Rapin et al. 2019; Fairén et al. 2023). Alkaline lakes in particular have received heightened attention in recent years due to, among their many attributes (Chase et al. 2021; Raudsepp et al. 2023; Tutolo and Tosca 2023), a propensity to accumulate vital ingredients for leading origin‐of‐life hypotheses (Toner and Catling 2019, 2020), their unusually high primary productivity compared to other ecosystems (e.g., Jones et al. 1998), and their capacities to show extreme stable isotope enrichments (Stüeken et al. 2015, 2020; Tino et al. 2023). The latter may be a way to identify these systems in the rock record.

The elevation of (1) dissolved inorganic carbon, (2) salinity, and (3) pH—at least under the Earth's current atmospheric *p*CO_2_ (Hurowitz et al. 2023)—are primary attributes that distinguishes alkaline lakes from ordinary freshwater systems. Previous studies of nitrogen availability in alkaline lakes have primarily focused on the effects of high pH on nitrogen chemistry. At pH 9.25, ammonia [NH_3_] and ammonium [NH_4_^+^] are present in equal parts in solution (p*K*_a_ = 9.25, at standard conditions). As pH approaches and/or exceeds this value, a greater proportion of NH_3_ volatilizes out of solution. This relationship favors volatilization of ^14^N, imparting ẟ^15^N fractionations of > 40‰ on the escaping NH_3_ and leaving the residual NH_4_^+^ isotopically heavy (Li et al. 2012). While this fractionation decreases in magnitude with increases in temperature, it is still as high as approximately 33‰ at 70°C (Li et al. 2012). Values of ẟ^15^N ≥ 10‰ in sediments from evaporitic lakes have therefore been used as indicators of elevated pH during the time of deposition (Collister and Hayes 1991; Talbot and Johannessen 1992; Menzel et al. 2013; Stüeken et al. 2015, 2020). Yet not all high‐pH settings consistently exhibit this phenomenon (e.g., Muzuka et al. 2004; Xu et al. 2006), and the potentially overlapping isotope effects of denitrification, which is redox‐dependent (Hecky et al. 1996) but not directly pH‐dependent, must always be considered. Therefore, an interplay of the multiple parameters associated with alkaline lakes, including but not exclusively pH, appears to be responsible for enabling elevated ẟ^15^N in the rock record (Xia et al. 2022). Developing a more thorough understanding of these complexities is important for accurate applications of the ẟ^15^N proxy to ancient sedimentary strata.

Here we leverage the Coorong region of South Australia as a natural laboratory to interrogate the effects of alkalinity and evaporation (i.e., hypersalinity) with respect to not only the generation of high ẟ^15^N signals but also their long‐term preservation. Key topics include (1) the balance of abiotic and biological controls on nitrogen speciation and potentially ammonia volatilization; (2) which stable isotopic systems can best inform aqueous restriction, and how varying degrees of restriction may alter the effectiveness of these proxies; and (3) whether principal component analyses can elucidate otherwise cryptic aspects of the N‐cycling and proxy preservation in alkaline‐hypersaline settings when multiple data types are available. Our evaluations of restriction are targeted at determining sites and/or intervals of closed‐basin behavior, in which evaporation is the dominant form of water removal (i.e., significantly exceeding groundwater seepage and within an environment lacking an evident outlet [endorheic]). This evaluation will be done primarily through examinations of multiple conventional stable isotope systems (ẟ^13^C_carb_ and ẟ^18^O_carb_), bulk sedimentary chemical abundances (total organic and inorganic carbon [TOC and TIC], and salinity‐associated elements [Na, Sr, and K, among others]). Collectively, these data provide context for interpreting the bulk ẟ^15^N record (here measured on decarbonated sediments to concentrate the N content of organic matter and potentially clays). Importantly, among our primary goals is an improved understanding of where and when elevated ẟ^15^N signals best survive early diagenesis.

## 2 Site Description

**FIGURE 1:**
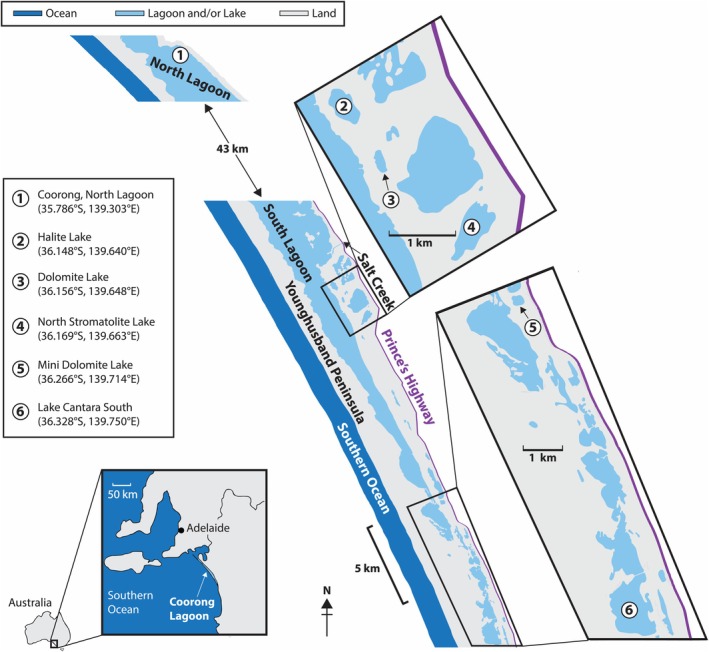
Geographical context for the studied portion of the Coorong region, including the locations of all field sites in this study. Adapted from Wright (1999).

The Coorong region in South Australia (Figure 1) hosts several ephemeral lakes that span both an existing and historic pH gradient (von der Borch 1965; De Deckker and Geddes 1980; Wright 1999; Wright and Wacey 2005). Consequently, this area is well suited for determining which aspects of nitrogen cycling are most affected by pH and evapo‐concentration and, in turn, for establishing links to persistent and preservable ẟ^15^N enrichments if present. The Coorong is broadly defined by a shallow lagoon system that is separated from the Southern Ocean by the Younghusband Peninsula. It runs shore‐parallel to the South Australian coastline for over 130 km (Webster 2010). The Coorong is a national park, a Ramsar‐listed conservation site, and part of the Traditional lands of the Ngarrindjeri people. Anthropogenic chemical inputs are minimal. As a “choked” lagoon, its narrow entryway effectively mutes the impact of tidal oscillations on water level fluctuation (Kjerfve 1994). Over the past approximately 90 years, salinity has risen substantially, reaching over 15% (or > 150 practical salinity units [PSU]) during the early austral autumn in the South Lagoon (Webster 2010). The changing conditions are tied in part to the construction of dams in the 1930s, emplaced to prevent ocean waters from mixing with the nearby Lake Alexandrina (Wright and Wacey 2005). These dams have exacerbated the effect of seawater input being less than the evaporation rate, particularly in the more‐restricted, southern portion of the lagoon (Webster 2010).

Several ephemeral lakes of varying biogeochemical compositions exist within approximately 2 km of the permanent lagoon itself (Figure 1). Most of these lakes occupy interdunal furrows within a series of Pleistocene beach‐dune ridges that formed during interglacial high stands over the last several hundred thousand years (Warren 1988). Historically, these systems have been the subject of studies detailing the active, primary formation of dolomite [CaMg(CO_3_)_2_] (Mawson 1929; Rosen et al. 1988; Warren 1988, 1990; Wright 1999), which is rare on the modern Earth but forms in abundance in the Coorong lakes and is frequently present in the sedimentary rock record (Land 1985). These lakes also contain Mg‐carbonates (e.g., magnesite and hydromagnesite), a topic of interest in Mars research due to the apparent presence of these minerals at the Martian surface (Ehlmann et al. 2008), including within Jezero crater (Horgan et al. 2020; Zastrow and Glotch 2021). The ephemeral lake waters within the Coorong region are fed by a combination of meteoric input (i.e., groundwater and precipitation) and seawater intrusions (Rosen et al. 1988, 1989; Shao et al. 2018). Thus, diverse lake chemistries occur as a result of variations in both freshwater–seawater mixing and evaporation rates (Warren 1990; Shao et al. 2018). It is this geochemical variation that we aim to leverage as a testbed for nitrogen cycling dynamics under extreme conditions.

In previous literature, the sedimentary package (approximately 5–10 m depth) below the lakes was subdivided into four informal units (Warren 1988). The bottom two units (termed *basal* and the overlying *organic*‐*rich*) vary in composition laterally as a function of a specific area's connectedness to the open ocean during the Pleistocene (Warren 1988, 1990). Based on their relatively high siliciclastic contents (and thus lower carbonate contents), these units may not consistently reflect alkaline and hypersaline conditions and are therefore not the subject of this study. The *basal* and *organic*‐*rich* units are overlain by the so‐called *pelletal laminated* unit, recording a time when the lakes were perennially subaqueous (Warren 1988, 1990). The focus of our study is on the topmost or so‐called *massive* unit, which sits directly below modern surface waters, is typically 40–60 cm thick, and contains most of the Holocene dolomite in the region (Rosen et al. 1988; von der Borch 1976). The *massive* unit is comprised of calcareous mudstones, wackestones, and pelletal packstones in a manner that is related to the energy of the overlying waters, with mudstones depositing in the deepest, lowest energy settings and packstones characterizing the higher energy margins (with wackestones at intermediate depths) (Warren 1988).

The three northernmost lakes in this study (Halite, Dolomite, and North Stromatolite) are part of the Salt Creek lake chain (Figure 1). This area developed as recently as the early Holocene, when a narrow estuarine lagoon branched off of the greater lagoon and was subsequently isolated by the development of a sandy ridge (Warren 1990). Moving southward, the permanent lagoon eventually ends and becomes a series of ephemeral lakes. Mini Dolomite Lake lies a few kilometers north of where that transition occurs. All four of the above‐mentioned lakes sit atop calcrete‐floor depressions within the same approximately 120 kya interdunal corridor (classified by Warren 1990, as “Type 2a”). The fifth lake in this study, Cantara South Lake, resides even farther southward and formed during a more recent breakup of the current lagoon system (classified as “Type 2b” in Warren 1990). Previous studies have indicated that the *massive* unit of each lake in this study area differs from the others in terms of its carbonate mineral assemblages (e.g., Warren 1990; Raudsepp et al. 2022). This variation informed our sampling strategy because we aimed for geochemical diversity in our examination of the differing controls on nitrogen cycling.

## 3 Methods

### 3.1 Sample Collection

Sediment cores were collected over two days in July 2018 (July 7 and 8) during the early austral winter and a period of significant rainfall. As discussed below, this rainfall may have impacted the utility of the top‐most sediment in each core. Five lakes were chosen with the intent of capturing a heterogeneous distribution of near‐shore lake chemistry. The locations are, from north to south (Figure 1): Halite Lake (36.148° S, 139.640° E), Dolomite Lake (36.156° S, 139.648° E), North Stromatolite Lake (36.169° S, 139.663° E), Mini Dolomite Lake (36.266° S, 139.714° E), and Lake Cantara South (36.328° S, 139.750° E). An additional core was taken from the North Lagoon (36.169° S, 139.663° E) to test whether our sampling and geochemical analyses produced results in line with previous studies. The pH of the surface waters in each sampling location was measured with a YSI professional Plus pH‐ORP dual‐sensor, although we note that the heavy rain at that time may compromise the utility of these data. Push cores were composed of clear, rigid polycarbonate tubing (2.75" outer diameter, 2.50" inner diameter). Each core was sliced into 2 cm sample increments. These were homogenized, transferred to 50 mL conical Falcon tubes, and frozen. Samples were freeze‐dried, ground into dry powders with an agate mortar and pestle, and stored in scintillation vials prior to geochemical analyses.

### 3.2 Geochemical Analyses

Total organic carbon [TOC] and total inorganic carbon [TIC] data were collected in the Lyons Biogeochemistry Laboratory at the University of California, Riverside [UCR]. Total Carbon [TC] was measured via combustion, and TIC was measured via acidification, both using an Eltra CS‐500 carbon sulfur analyzer (with a precision better than ±0.1 wt%; TOC and TIC values measured as < 0.1 wt% are reported as ≤ 0.1 wt%). TOC was then calculated as the difference between the measured TC and TIC.

All stable isotope ratios in this study were measured via continuous flow systems. For isotopic analyses on decarbonated samples (δ^15^N and δ^13^C_org_), 0.5–5 g of powder were treated with 1 N HCl at 50°C overnight and washed three times with 18 MΩ cm^−1^ DI‐H_2_O (a variation of Stüeken et al. 2015; Song et al. 2023). The dried residues were weighed into tin capsules and analyzed at the University of St Andrews on an EA Isolink coupled to a MAT253 isotope ratio mass spectrometer (IRMS) via a Conflo IV. This process also yielded total nitrogen (TN_decarb_ wt%) and C/N (mol/mol) of the decarbonated materials. The δ^15^N and δ^13^C_org_ data were calibrated with international reference materials USGS‐40 and USGS‐41 and displayed a precision of ±0.47‰ or better for samples analyzed in duplicate, except in the case of the North Lagoon sample “NL8,” which yielded ±1.15‰ for δ^15^N. Sample “NS12” is not reported in this study due to near‐zero TN_decarb_. Values are reported in standard delta notation relative to Vienna Pee Dee Belemnite (V‐PDB) for δ^13^C_org_ and relative to air (AIR) for δ^15^N.

Stable isotopes of carbonates (δ^18^O_carb_ and δ^13^C_carb_) were analyzed at UCR by reacting with 104% phosphoric acid (based on specific density; Burman et al. 2005) at 50°C in a Thermo Scientific GasBench II device, followed by CO_2_ analysis with a Delta V Advantage IRMS. It is well established that this routine acidification step yields differing fractionation factors with respect to δ^18^O_carb_ in calcite [CaCO_3_] versus dolomite. Therefore, two sets of data were generated: one that assumed an exclusively dolomitic composition of carbonates and another that assumed an exclusively calcitic composition. A weighted average of the two values was taken based on Ca and Mg contents determined via ICP‐MS to generate the reported data. Two calibration standards, NBS18 and NBS19, were used in the same analytical run. The precision was 0.1‰ for δ^13^C_carb_ and 0.2‰ for δ^18^O_carb_ as reported relative to V‐PDB and Vienna Standard Mean Ocean Water [V‐SMOW], respectively.

Bulk element abundances were measured via inductively coupled plasma mass spectrometry (ICP‐MS) at the University of St Andrews. Approximately 70 mg of each powdered sample were weighed into an acid‐washed 15 mL centrifuge tube and mixed with 6.93 mL of 10% (*v*/*v*) trace‐metal grade HNO_3_. The samples were left to react for 2 h at room temperature. During that time, they were shaken manually every ca. 20 min. After 2 h, the caps were loosened, and the centrifuge tubes were placed into an acid‐proof oven at 70°C for 1 h. Next, the caps were retightened, and the samples were centrifuged at 3900 rpm for 15 min. From the supernatant, 50 μL were extracted with a pipette and mixed with 4.95 mL of 5% HNO_3_ in a fresh centrifuge tube. Prior to analysis, 30 μL of each diluted sample were mixed with 1.47 mL of 2% HNO_3_ inside an acid‐washed 2 mL centrifuge tube. Analyses were carried out with an Agilent 7500 ICP‐MS equipped with a Teflon spray chamber. Multi‐element calibration standards were run at the start, in the middle, and at the end of the run to monitor and correct for drift. Reproducibility (relative error) for all elements was better than 12% and often better than 5%.

### 3.3 Principal Component Analyses (PCA)

Principal component analyses were executed in the programming language R using the integrated development environment RStudio. PCA is a commonly used statistical approach with applications in many disciplines, including stable isotope ecology. For fundamental information on PCA, see Meglen (1992) and Bro and Smilde (2014). In all PCAs in this study, the data are overlain by color‐coded ellipses that represent the 95% confidence interval. The rationale for the variety of executed PCAs in this study is provided in Section 5.4.

## 4 Results

**FIGURE 2:**
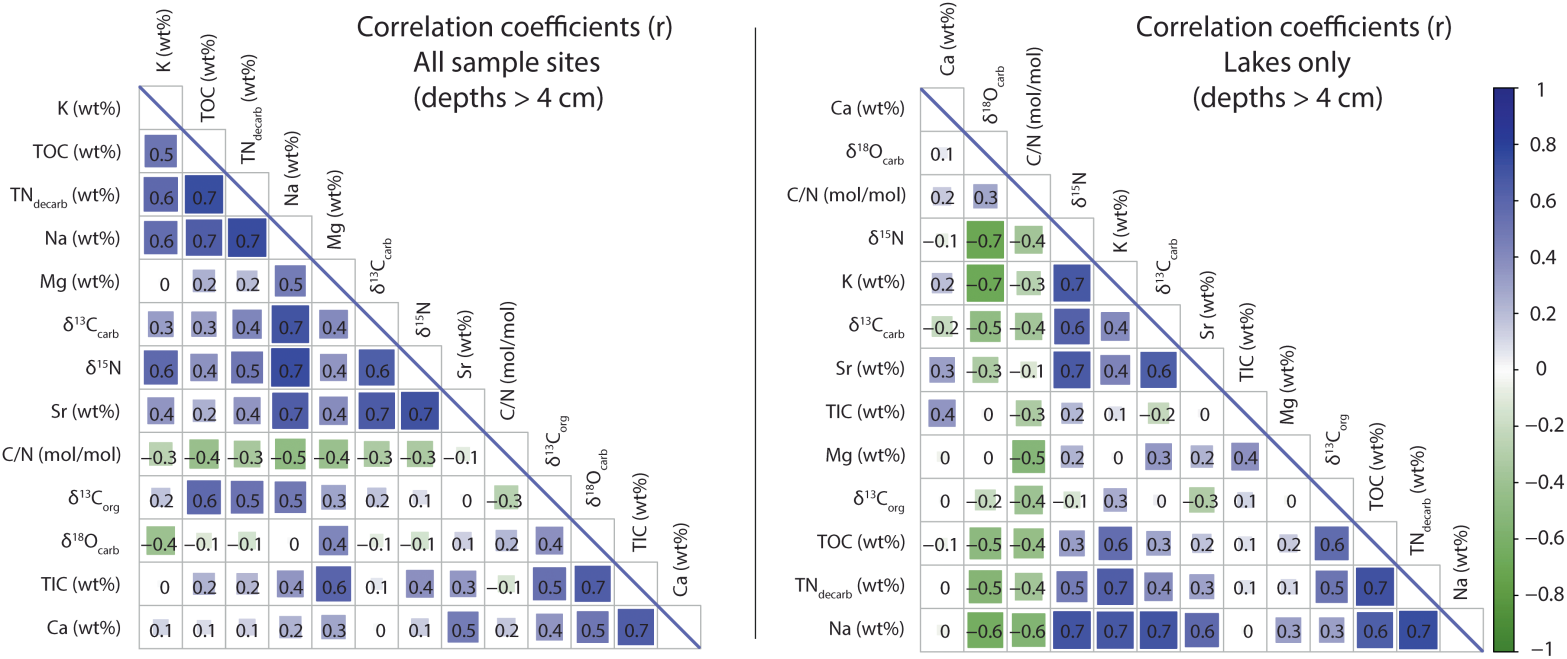
Correlation plots for the 13 different geochemical parameters measured in this study. Only sample depths > 4 cm are included in this plot; this filter tends to increase Pearson correlation coefficients (*r*) because it may serve to eliminate inconsistencies caused by high microbial respiration rates and/or rainwater dilution near the surface.

All stable isotope measurements are reported in Table S1; abundances and ratios are listed in Tables S2 and S3. Figure 2 provides correlation plots of all reported variables at sampling depths > 4 cm. Filtering data by this sample depth tends to increase Pearson correlation coefficients (*r*). We suspect that the uppermost 4 cm display inconsistencies caused by recent rainwater dilution and high microbial respiration rates (see Section 5). For the rest of the study, we therefore focus on sediments at > 4 cm depth, as long‐term preservation of biogeochemical signals is the focus of this study.

### 4.1 Stable Nitrogen and Carbon Isotopes of Decarbonated Sediments

**FIGURE 3:**
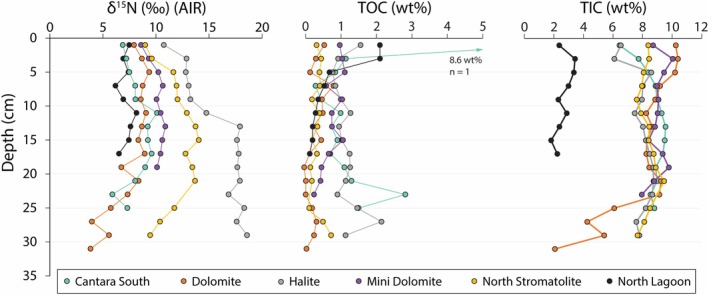
Geochemical trends (δ^15^N, TOC, and TIC) with depth at all study sites. Individual sites are distinguished by color‐coding. There is high intersystem variability in δ^15^N. The North Lagoon site (black) functions as an endmember because it is not a lake and is fed directly by the open ocean.

Across all sites, δ^15^N ranges from 3.8‰ to 18.6‰ (Figure 3), while δ^13^C_org_ spans −25.7‰ to −16.4‰. The δ^15^N data exhibit a narrower range in the top 4 cm of sediment, potentially due to isotopic dilution via meteoric nitrate introduced by heavy rainfall during the sampling period. At North Lagoon (mean δ^15^N = 7.2‰, *n* = 9, *σ* = 0.6‰), which serves as an end member because it is fed directly by the open ocean with only minor freshwater input, values span from 6.2‰ to 8.2‰. This range is slightly enriched relative to the average modern marine nitrate (NO_3_^−^) value of approximately 5‰ but lower than values reported from marine upwelling zones (9‰–12‰) (Tesdal et al. 2013). Carbon isotope data of the decarbonated materials (δ^13^C_org_) at North Lagoon are invariant (mean = −25.1‰, *n* = 9, *σ* = 0.4‰) and the lowest of all sample sites.

Halite Lake (mean δ^15^N = 15.8‰, *n* = 15, *σ* = 2.5‰) exhibits the highest δ^15^N; values broadly increase with depth before reaching a maximum of 18.6‰ at the base of the core (28–30 cm). Carbon isotope values at Halite are, on average, the second highest of any site (mean δ^13^C_org_ = −20.0‰, *n* = 15, *σ* = 0.6‰). North Stromatolite is the only other system to consistently display δ^15^N > 11‰ (mean = 11.9‰, *n* = 15, *σ* = 1.6‰), with an apparent trend that starts at 9.0‰ and increases with depth until reaching 14.0‰ at 14–16 cm before descending back to 9.5‰ at the bottom of the core. The δ^13^C_org_ data at that site (mean = −23.4‰, *n* = 15, *σ* = 1.1‰) are the lowest of any sampled lake, with a minimum of −25.7‰ at 6–8 cm before consistent increases lead to a maximum of −21.7‰ at the bottom of the core. Dolomite Lake (mean δ^15^N = 7.5‰, *n* = 16, *σ* = 1.7‰) shows δ^15^N data that increase from 7.9‰ at the surface to a maximum of 9.4‰ at 4–6 cm depth. Values persist in the range of 6.7‰ to 9.1‰ before decreasing to 5.7‰ at 24–26 cm and eventually reach a minimum of 3.8‰ at 30–32 cm. The δ^13^C_org_ values at Dolomite (mean = −21.4‰, *n* = 16, *σ* = 0.7‰) range between −22.8‰ to −20.5‰, in line with the average value of all lake samples (mean = −20.8‰, *n* = 68, *σ* = 1.9‰). At Cantara South (mean δ^15^N = 8.2‰, *n* = 13, *σ* = 1.2‰), δ^15^N increases from 6.9‰ at the surface to a maximum of 10.1‰ at 10–12 cm depth; values are ≥ 9‰ until 20 cm and then fall to a minimum of 5.9‰ at 22–24 cm. The δ^13^C_org_ data at Cantara South are the highest of any site (mean = −18.1‰, *n* = 13, *σ* = 0.9‰). Mini Dolomite Lake (mean δ^15^N = 10.1‰, *n* = 10, *σ* = 0.7‰) has δ^15^N = 8.6‰ at the surface followed by data that are clustered between 10.0‰ and 10.9‰ for the remainder of the core. The carbon isotopes there fit within a small range between −19.7‰ and − 22.1‰ (mean δ^13^C_org_ = −21.4‰, *n* = 10, *σ* = 0.7‰).

### 4.2 Total Organic and Inorganic Carbon, Total Nitrogen, C/N Ratios

Total organic carbon is either uniformly below 2 wt% or tends to exceed that value only near the sediment–water interface. The North Lagoon contains 2.1 wt% TOC from 0 to 4 cm, followed by an abrupt decrease to 0.7 wt% at 4–6 cm and eventually a minimum of 0.1 wt% at the bottom of the core. Cantara South possesses the highest TOC, both at the surface (7.3 wt% at 0–2 cm depth) and overall (mean = 1.5 wt%, *n* = 13, *σ* = 1.8%). Halite Lake has the second highest average TOC (mean = 1.2 wt%, *n* = 15, *σ* = 0.3%) and is notable for its relatively consistent values that range from 0.8 wt% to 2.1 wt%. Dolomite and North Stromatolite have uniformly low TOC contents (≤ 0.8 wt% in all samples), while Mini Dolomite is elevated by comparison (mean = 0.8 wt%, *n* = 10, *σ* = 0.2%).

These historically carbonate‐bearing sediments have TIC values that are elevated compared to most terrestrial or marine settings. Average TIC contents, from highest to lowest, are: Mini Dolomite (mean = 9.2 wt%, *n* = 10, *σ* = 0.5%), Cantara South (mean = 8.7 wt%, *n* = 13, *σ* = 0.8%), North Stromatolite (mean = 8.3 wt%, *n* = 15, *σ* = 0.5%), Dolomite (mean = 7.9 wt%, *n* = 15, σ = 2.2%), and Halite (mean = 7.9 wt%, *n* = 15, *σ* = 0.8%). For reference, TIC in pure calcite and dolomite is 12.0% and 13.0%, respectively. Dolomite Lake displays a decrease from 9.1 wt% to 2.1 wt% over the deepest 10 cm of core. This decrease coincides with a 3.5‰ decrease in δ^15^N. The North Lagoon contains significantly less TIC compared to the other studied lakes (mean = 2.6 wt%, *n* = 9, *σ* = 0.5%).

The total nitrogen content (TN_decarb_) correlates with TOC (*R* ^2^ = 0.42, *n* = 77, *p* < 0.001) across all samples, indicating that most of the nitrogen analyzed is preserved within organic matter. Some proportion of TN_decarb_ is also likely sourced from clay‐bound N formed during diagenesis (Müller 1977), but the isotopic consequences of this process are small (< 2‰; Robinson et al. 2012, Li et al. 2021). Additionally, the high salinity of the Coorong study sites may diminish the amount of clay‐bound N due to the potential role of alternative cation substitution in clays (Rysgaard et al. 1999). The moderate correlation (*r* = 0.65, *p* < 0.001) between TN_decarb_ and TOC indicates that N is predominantly biomass‐derived, and detrital clay‐bound N is minimal. Halite Lake displays the highest average TN_decarb_ value (mean = 0.56 wt%, *n* = 15, *σ* = 0.37 wt%), including a maximum of 1.67 wt%. Cantara South (TN_decarb_ mean = 0.40 wt%, *n* = 13, *σ* = 0.36 wt%) exhibits elevated TN_decarb_ contents (1.18 wt%) at the surface before decreasing to a minimum of 0.03 wt% at 8–10 cm depth. TN_decarb_ is uniformly low (≤ 0.22 wt%) at the Dolomite, Mini Dolomite, North Stromatolite, and North Lagoon sites. The following are the average C/N ratios of those same decarbonated materials (mol/mol, where C is short for TOC), from least to greatest: Halite (mean = 13.6, *n* = 15, *σ* = 2.5), North Lagoon (mean = 15.6, *n* = 9, *σ* = 1.6), Mini Dolomite (mean = 16.2, *n* = 10, *σ* = 1.5), Cantara South (mean = 16.8, *n* = 13, *σ* = 2.7), Dolomite (mean = 18.2, *n* = 16, *σ* = 3.4), and North Stromatolite (mean = 19.3, *n* = 15, *σ* = 2.4). Notable observations include increasing C/N with depth at the Halite, Dolomite, and North Stromatolite sampling sites.

### 4.3 Bulk Element Abundances via ICP‐MS

**FIGURE 4:**
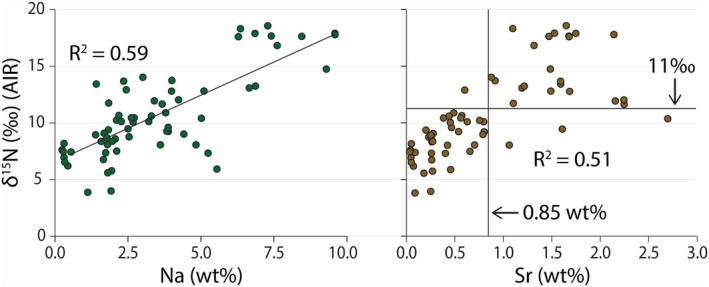
Scatterplots of δ^15^N data versus Na and Sr, from depths > 4 cm. There is a statistically moderate relationship between both δ^15^N versus Na (wt%) and δ^15^N versus Sr (wt%). There is also an apparent threshold in the δ^15^N versus Sr (wt%) data, where 96% of samples with δ^15^N > 11‰ also have Sr > 0.85 wt%.

While high Ca and Mg values can be expected in these known Ca‐ and Mg‐bearing carbonate sediments, all lake sites are also high in Na, K, and Sr (henceforth grouped as salinity‐associated elements [SAE]). Generally, TN_decarb_ is correlated with Na and K to the same extent (Figure 2), which implies that K is mostly salt‐bound as opposed to clay‐bound. There are significant differences in SAEs among the studied lake sites. For example, the difference between the highest average Na content at Halite (mean = 7.40 wt%, *n* = 15, *σ* = 1.45%) and the lowest at Dolomite (mean = 1.70 wt%, *n* = 16, *σ* = 0.37%) is 6.7 wt%. The other three lakes span a middle range: Mini Dolomite (mean = 2.43 wt%, *n* = 10, *σ* = 0.74%), North Stromatolite (mean = 3.08 wt%, *n* = 15, *σ* = 1.06%), and Cantara South (mean = 3.77 wt%, *n* = 13, *σ* = 1.39%). The δ^15^N values across all sites correlate with Na (*R* ^2^ = 0.53, *p* < 0.001) and Sr (*R* ^2^ = 0.46, *p* < 0.001), and coefficients of determination increase (*R* ^2^ = 0.59, *p* < 0.001 for Na and *R* ^2^ = 0.51, p < 0.001 for Sr; Figure 4) when considering depths > 4 cm only.

### 4.4 Stable Carbon and Oxygen Isotopes of Carbonates

There are no extreme depletions or enrichments in δ^13^C_carb_ (ranging from −3.2‰ to 5.0‰) or δ^18^O_carb_ (ranging from 30.6‰ to 37.3‰) at any site. The North Lagoon samples approximate values expected from a marine environment (δ^13^C_carb_ mean = −0.4‰, *n* = 9, *σ* = 0.7‰; δ^18^O_carb_ mean = 31.9‰, *n* = 9, *σ* = 0.6‰). All lake sites show elevated values by comparison, except for δ^13^C_carb_ at Dolomite, which is lower (mean = −1.2‰, *n* = 16, *σ* = 1.3‰). Conversely, Dolomite Lake contains the highest δ^18^O_carb_ (mean = 36.1‰, *n* = 16, *σ* = 0.4‰), while Halite has the highest average δ^13^C_carb_ by a margin of 1.7‰ (mean = 3.4‰, *n* = 15, *σ* = 1.3‰).

**FIGURE 5:**
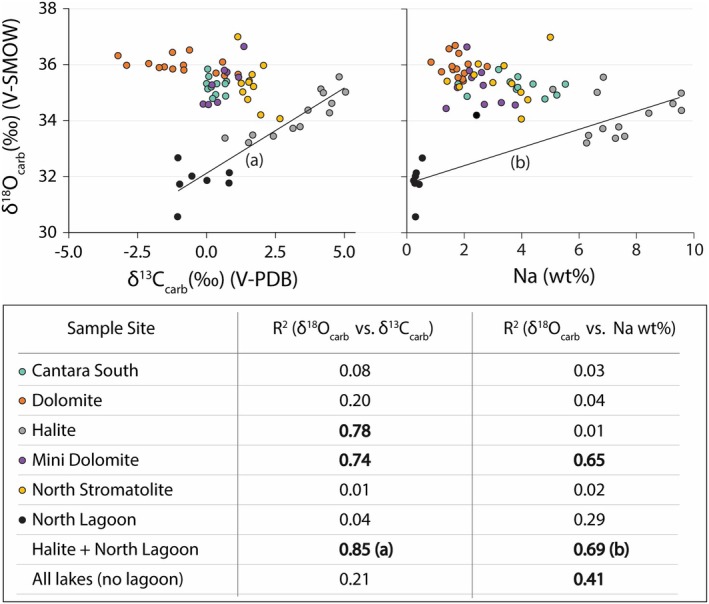
(Top) Scatterplots of δ^18^O_carb_ versus δ^13^C_carb_ and δ^18^O_carb_ versus Na wt%, from depths > 4 cm. (Bottom) Coefficients of determination for the variables of interest at each sample site, as well as across all lakes. (a, b) Halite Lake and North Lagoon display a statistically strong relationship in δ^18^O_carb_ versus δ^13^C_carb_ space, indicating that Halite Lake may be evolved from a seawater source. This is in agreement with previous sedimentological and mineralogical evidence (Warren 1990).

Coupled δ^13^C_carb_ and δ^18^O_carb_ data from carbonates (Figure 5) show correlation in only two systems: Halite (*R* ^2^ = 0.78, *p* < 0.001) and Mini Dolomite (*R* ^2^ = 0.74, *p* < 0.05). Mini Dolomite also displays a relationship between Na and δ^18^O_carb_ (*R* ^2^ = 0.65, *p* < 0.05). When Halite and North Lagoon samples are grouped, relationships are apparent between δ^13^C_carb_–δ^18^O_carb_ (*R* ^2^ = 0.85, *p* < 0.001) and Na–δ^18^O_carb_ (*R* ^2^ = 0.69, *p* < 0.001). Across all lake systems, there is a moderate relationship between Na and δ^18^O_carb_ (*R* ^2^ = 0.41, *p* < 0.001).

## 5 Discussion

### 5.1 Geochemistry of the Coorong Lakes

A high degree of variation in δ^15^N exists at the studied lakes (Figure 3), despite their proximity to one another and several first‐order geologic similarities. These systems have likely been alkaline and shallow (cm‐scale water column, excluding Cantara South) for at least several hundred years, including the interval captured by our data (i.e., the *massive* unit of Warren 1988; see Raudsepp et al. 2022 for radiocarbon dating). Thus, the differences in sedimentary chemistry observed among the studied lakes may offer direct insights into which geochemical parameters are connected to the preservation of elevated δ^15^N. When the lakes are ordered from lowest to greatest δ^15^N, they show a gentle gradient and, by extension, a framework for discussing δ^15^N‐amplifying processes that might otherwise be elusive. In particular, any of the observed trends would likely be more difficult to detect with an approach that focuses on only one lake.

### 5.2 Linkages Between Nitrogen Cycling and Salinity

The link between δ^15^N and SAEs (Figures 2 and 4) is strong in the Coorong lake sediments, motivating our discussion of the possible effects of salinity on nitrogen cycling. Importantly, there is extensive independent evidence that the stability and abundance of reduced nitrogen (e.g., NH_4_^+^) increase with salinity in hypersaline lakes, solar salterns, experimental cultures, estuaries, and the Coorong Lagoon itself, all under oxidizing conditions (Post 1977; Koops et al. 1990; Rysgaard et al. 1999; Isaji et al. 2019; Priestley et al. 2022; Mosley et al. 2023). This accumulation of reduced N, even under oxidizing conditions, may have multiple causes. At extremely high salinities (> 100 PSU), this relationship is perhaps most parsimoniously explained by physiological salinity limits of nitrifying bacteria (i.e., those that oxidize reduced nitrogen to nitrogen oxide species [NO_*x*_] as a means of metabolism) (Koops et al. 1990; Oren 1999). At salinities ranging from 35 to 100 PSU, lower rates of nitrification may be related to the well‐established relationship between increased salinity and decreases in dissolved O_2_ solubility (e.g., Garrels and Christ 1965). However, Rysgaard et al. (1999) documented in Randers Fjord estuary in Denmark that even salinity shifts in the range of 0–30 PSU may present physiological impairment of microbial nitrification within sediments. This impairment would act in concert with the observation that prevalent cations (e.g., Na^+^) can replace NH_4_^+^ in clays and thus reduce NH_4_^+^ adsorption and lead to sedimentary efflux of ammonic species into the water column. The net consequence of these two effects is enhanced ammonium buildup in the water column and potential release of NH_3_ to the atmosphere.

The effects of hypersalinity on nitrifying bacteria have been experimentally quantified. Perhaps the most straightforward example is Koops et al. (1990), where three *Nitrosomonas* species (obligate NH_*x*_ oxidizers; *N. halophilus, N. mobilis*, and *N. oceanus*) were isolated from hypersaline environments. Salt (Na‐Cl type) tolerances varied, but the highest tolerance was seen with *N. halophilus* , which exhibited optimum nitrification rates at 700 mM NaCl (40.9 PSU). Importantly, as 1700 mM NaCl (99 PSU) was approached, NO_2_^−^ production by nitrification was significantly diminished to near‐zero. These observations are in strong agreement with classic observations of the complete absence of NO_*x*_ in large portions of Great Salt Lake (Utah, USA; Post 1977) and a dearth of it (maximum of 20 μg/L) in the Dead Sea (Jordan Rift Valley; Nissenbaum 1975) even under oxic conditions in both cases.

How hypersalinity influences δ^15^N preservation in sediments (i.e., nitrogen bound to buried organic matter and possibly clays) is not as well studied. To interpret elevated isotope ratios found in solar salterns (Trapani, Italy), Isaji et al. (2019) presented a model of residually increased surface δ^15^N. Specifically, progressive ammonium assimilation in the subsurface may isotopically enrich surface ammonium (δ^15^N ≈ 34.0‰ in the surface brine). At a certain depth, the respiration of organic matter liberates organic N (e.g., R‐NH_2_) as NH_4_^+^. A series of microbial primary producers then assimilate the ammonium in a fashion that preferentially uptakes ^14^N over ^15^N and leaves ^15^N‐enriched ammonium to interact with the surface environment. At that point, evapo‐concentration of the hypersaline waters aids in the volatilization of NH_3_.

There are at least two critical distinctions between the systems described above and those of this study. Specifically, Coorong lake sediments have much greater carbonate contents and historically higher measured pH values, with some intervals reaching at least a pH of 9.26 and theoretically up to 10.20 based on mineralogical constraints (von der Borch 1965; Wright 1999). Previous studies have shown that the diagenesis of organic matter under oxic conditions can increase the measured δ^15^N value by a few permil, while anoxic diagenesis may decrease it (Freudenthal et al. 2001; Lehmann et al. 2002) or leave it unchanged (Busigny et al. 2024). In our study sites, the sediments are likely anoxic at shallow depths (see Wright and Wacey 2005), and therefore the observed down‐core increase in δ^15^N is unlikely to be a diagenetic artifact.

### 5.3 Assessing Closed‐Basin Behavior and Its Relationship With δ^15^N Enrichment

It is critical to evaluate for closed‐basin behavior at each study site, as this informs the potential for unidirectional escape of gaseous NH_3_ that outbalances total N input. This scenario would allow for heavily skewed residual δ^15^N at a given lake. The positive covariation of δ^13^C_carb_ and δ^18^O_carb_ is a common indicator for closed‐basin lacustrine systems (Talbot 1990). This relationship occurs because residual δ^18^O_carb_ is driven up by evaporation of H_2_O, while CO_2_ degasses due to corresponding increases in salinity, which generates a δ^13^C_carb_ isotope effect resulting from the preferential loss of ^12^C. By that measure, δ^13^C_carb_–δ^18^O_carb_ covariation evidence of closed‐basin behavior is surprisingly infrequent across our study sites. Only Halite and Mini Dolomite lakes display the expected δ^13^C_carb_–δ^18^O_carb_ correlation (Figure 5). This lack of δ^13^C_carb_–δ^18^O_carb_ covariation in the other lakes is surprising because they currently appear to be closed basins. Specifically, North Stromatolite and Dolomite are ephemeral on annual timescales (i.e., they undergo desiccation, which should increase isotopic covariation with salinity increases), and Cantara South does not show evidence of an outlet (i.e., is endorheic).

Changes in water sourcing through time may explain the infrequency of δ^13^C_carb_–δ^18^O_carb_ correlation. The South Lagoon itself is known to receive a significant influx of brackish meteoric waters flowing towards the coast (≥ 40%; Shao et al. 2018). If this meteoric water source varies in its impact among the studied Lakes, it may have shifted δ^13^C_carb_–δ^18^O_carb_ relationships away from straightforward covariation in some of these settings. The sites with strong δ^13^C_carb_–δ^18^O_carb_ correlation (Halite and Mini Dolomite), in contrast, may be the result of relatively consistent water sources through time.

In any case, Halite Lake has the highest observed δ^15^N values and displays a clear history of closed‐basin behavior in its δ^13^C_carb_ and δ^18^O_carb_ data. Importantly, that lake likely evaporated from seawater and/or saline lagoon water, as opposed to freshwater (Figure 5, relationship [a]). This interpretation is in agreement with previous sedimentological and mineralogical evidence (Warren 1990) and may explain its extreme elevation in SAEs—Na and Sr in particular—presumably related to evapo‐concentration. While Halite and Mini Dolomite lakes are both restricted, only Halite exhibits δ^15^N > 11‰. A direct evaluation of the two systems within the context of a statistical approach may allow us to shed additional light on these relationships.

### 5.4 Evaluating the Value of Principal Component Analyses at the Coorong

**FIGURE 6:**
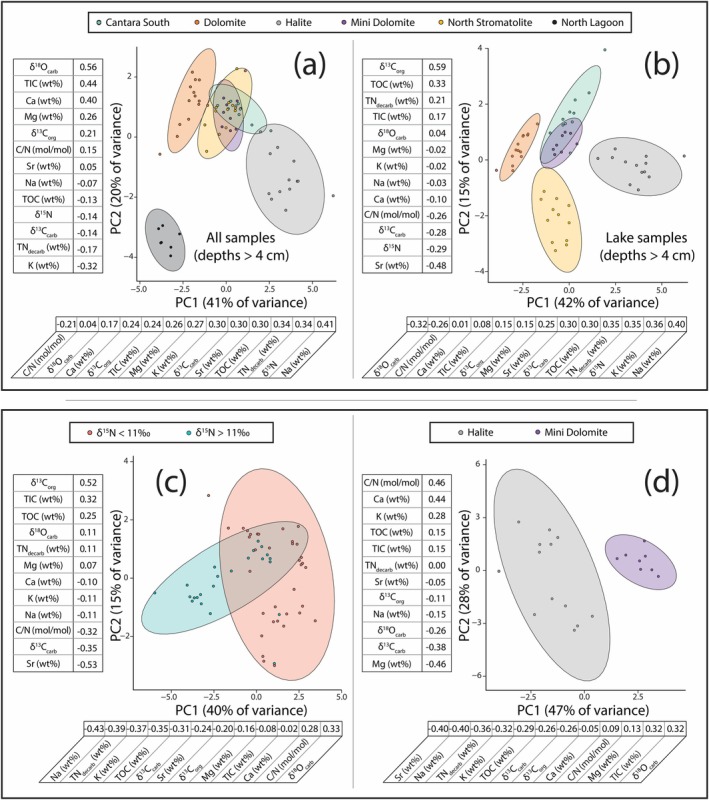
A stepwise series (a–d) of principal component analyses (PCAs). Color‐coded ellipses that represent a 95% confidence interval. The values placed above and below the *x*‐ and *y*‐axes—each associated with a specific variable—are loading scores. Loading scores are equivalent to the *r* value of a given variable versus its corresponding principal component value; they serve as a general indicator of which variables have the strongest influence on the position of a given data point in principal component space. The rationale and significance of each PCA are described in Section 5.4.

Principal component analysis (PCA) provides a means of visualizing and quantifying otherwise obscure but often meaningful trends within large datasets (Bro and Smilde 2014). We have taken a multi‐step approach resulting in four PCAs (Figure 6)—where one PCA informs the next—to elucidate drivers of elevated δ^15^N at the Coorong lakes. The first PCA (Figure 6a) takes all sample sites into consideration, including the North Lagoon. The inclusion of the North Lagoon end member serves to verify the approach, as the PCA noticeably separates this non‐lacustrine setting (bottom left of plot). The top loading scores (i.e., the *r* value of a given variable versus its corresponding principal component value) for PC1 indicate that δ^15^N and SAEs are the major discriminators in the dataset, once again validating the approach as this observation is functionally condensing information provided by more straightforward correlation and bivariate plots (e.g., Figures 2 and 4).

Removing the North Lagoon samples to create a lakes‐only PCA (Figure 6b) provides more insight. There is greater separation of Halite and Dolomite lakes from the other systems across PC1, establishing them as end members based primarily on differences in K, Na, δ^15^N, TN_decarb_, and δ^18^O_carb_. North Stromatolite lake, which possesses the second highest average δ^15^N (mean = 11.9‰, *n* = 15, *σ* = 1.6‰), is notably separate along PC2 due to its elevated Sr content. In the third PCA (Figure 6c), δ^15^N is removed as an input and the data are visually binned by whether δ^15^N is greater than or less than 11‰. This cutoff is established by (1) a boundary observed in our Sr vs. δ^15^N plot (Figures 2 and 4) a threshold that is not exceeded at Mini Dolomite Lake, despite strong evidence of being consistently endorheic through time (Figure 5). We also note that values greater than 11‰ are extremely rare in the marine realm (e.g., Tesdal et al. 2013), further highlighting that they represent a distinct hierarchy of biogeochemical and physicochemical processes. By changing δ^15^N from an input to a sampling bin, the aim is to determine which variables are most affecting nitrogen isotope fractionation. However, this PCA lacks the resolving power of the previous two (i.e., there is significant overlap between the two bins). One reason for this overlap may be the confounding nature of North Stromatolite Lake, which exceeds 11‰ at most depths but otherwise shows no history of being closed‐basin or having contributions of marine or lagoon water. Despite these ambiguities, North Stromatolite Lake does share one major similarity to Halite Lake (beyond δ^15^N > 11‰): high Sr compared to all other sites in this study. Thus, the process that led to elevated Sr contents may be related to the process that caused N isotope enrichment in these systems. The fourth PCA (Figure 6d) confirms this conclusion. Halite and Mini Dolomite show the greatest degree of closed‐basin behavior as manifest in C and O isotope trends, yet Mini Dolomite never exceeds δ^15^N > 11‰. The results of a PCA directly comparing the two (Figure 6d) indicate that Sr is as strong a differentiator as Na, and that Sr is more influential on δ^15^N than K. Moreover, the loading score of Sr has increased in absolute value from 0.25 and 0.24 in Figure 6b and Figure 6c, respectively, to 0.40 in Figure 6d.

Altogether, the PCA results provide at least two key insights. First, nitrogen chemistry is among the strongest differentiators in these systems. This may imply that elevated δ^15^N effectively functions as a proxy for hypersalinity in these systems. However, this relationship must be approached with caution because there is no obvious process by which high salinity alone would cause a significant increase in sedimentary δ^15^N. Salinity is likely one component of a more complex biogeochemical and physicochemical mechanism (see Section 5.5), which includes pH‐dependent ammonia volatilization as the major fractionation step. Near‐surface organic matter degradation in the sediments (to liberate ammonium) and endorheic behavior should also be indicated by independent lines of evidence because they are necessary for the accumulation of ammonic N in surface waters. Other factors that deserve consideration include the apparent need to surpass a certain salinity level (at least 100 PSU; see Section 5.2) and whether N is a limiting nutrient. Additionally, the PCAs indicate that a dissolved Sr threshold (here inferred from Sr wt% in the solid phase) may be the most critical inhibitor of nitrification. At Halite Lake, it appears that the elevated Sr wt% is a result of connectedness to marine‐derived waters (Figure 5a). The possible effects of Sr toxicity on nitrogen cycling (and on microbial nutrient cycling in general) have not been described previously to our knowledge, and thus our study points to a potentially fruitful new avenue of fundamental research.

### 5.5 A Proposed Mechanism for Elevated δ^15^N at Halite Lake

**FIGURE 7:**
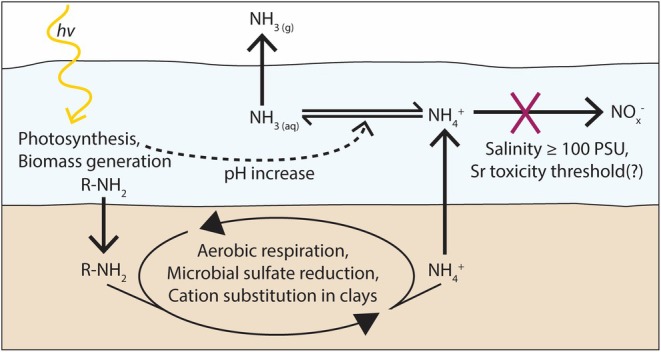
A proposed schematic of hypersaline nitrogen cycling that results in elevated δ^15^N. The mechanism is described in detail in Section 5.5 and is broadly adapted from Isaji et al. (2019), although there are several key differences. These differences include a holistic consideration of near‐surface organic matter degradation, cation substitution in clays, and the recognition of a possible Sr toxicity threshold.

We propose a mechanism for δ^15^N elevation that builds off previous literature while incorporating new insights from the Coorong lakes. Our conceptual model presented in schematic Figure 7 is similar to that of Isaji et al. (2019) but differs in multiple key ways, including specific considerations of near‐surface organic matter degradation and cation substitution in clays (e.g., Rysgaard et al. 1999; Wacey et al. 2007). First, photosynthesis results in biomass generation. The primary production in these lakes is driven by both cyanobacteria and algae. Cyanobacteria are evidenced by (1) a variety of stromatolitic features across these systems and suggestions from (2) biogenic dolomite morphologies observed under scanning electron microscopy (von der Borch 1976; Wright 1999). Evidence of algae includes C/N ratios measured in our study (Table S2), which in some cases align with those of algae from a regional ecology survey (C/N in the range of 10–20; Krull et al. 2009). Nitrogen is assimilated during photosynthesis and buried as biomass. Then, organic matter breakdown remineralizes assimilated N as ammonium and delivers it back to the water column (Berner 1980; Isaji et al. 2019). Microbial sulfate reduction (MSR) has been shown to occur within the uppermost sediment layers of some of these Coorong lakes (0–10 cm depth; Wacey et al. 2007), but sulfate reduction cannot easily be coupled to ammonium oxidation due to unfavorable thermodynamics. Hence, ammonium is generated during MSR and readily supplied back to the water column. We also posit that hypersalinity exacerbates ammonium release to the water column due to salinity‐associated cation substitution in clays at the sediment–water interface, as discussed above (Rysgaard et al. 1999).

Once formed, ammonium is relatively stable in the hypersaline water column even under oxic conditions (e.g., Isaji et al. 2019). Here we invoke a salinity threshold of 100 PSU, which is based on documented physiological limitations of hypersaline nitrifiers (approximately 99 PSU; Koops et al. 1990). Given that the south lagoon itself reaches > 150 PSU (Webster 2010), the ephemeral lakes are likely capable of exceeding this value during evapo‐concentration. For example, Halite Lake salinity was observed at 162 PSU in August 2018 by Shao et al. (2021). If the water column is above 100 PSU, then the conversion of NH_4_^+^ to NO_*x*_ is significantly diminished. Further, the pH of the waters then dictates the equilibrium chemistry of the reduced N‐species (i.e., as pH increases, a larger proportion of ammonium is converted to ammonia). The total amount of ammonia that is volatilized is likely influenced by various additional parameters such as temperature and salinity. It is important to note that the proposed mechanism applies to shallow water environments that are not redox‐stratified. Because the δ^15^N fractionation associated with denitrification is thought to be directly proportional to the amount of nitrate consumed within the water column (Kessler et al. 2014; Rooze and Meile 2016), it is unlikely to generate a significant N‐isotope expression in shallow, oxic hypersaline settings. Additionally, our model significantly hampers nitrate generation at > 100 PSU and, by extension, cuts off the oxidant supply (NO_*x*_^−^) needed for denitrification. Evaluations of deeper closed‐basin systems that display redox and/or salinity stratification would have to account for the potential effects of salinity gradients and water‐column denitrification on δ^15^N.

Notably, our field measurements (Table S4) did not display pH > 8.6 at any site (i.e., below the pKa of ammonium [9.25]). Halite Lake (with the highest sedimentary δ^15^N) yielded a pH value of 7.59. Importantly, however, pH fluctuations in the Coorong lakes are rapid and transient on sub‐seasonal timescales (Wright and Wacey 2005). For example, Raudsepp et al. (2022) measured Pellet Lake—which is within the Salt Creek area that includes the Halite, Dolomite, and North Stomatolite Lakes—at a pH of 10.0 in October 2016. This value is significantly higher than those recorded for the same system throughout November–early December by Wright and Wacey (2005) (pH ranging from 7.78 to 8.37). These fluctuations create difficulties when attempting to address current and historical pH maximums and minimums for each system. Our sediment cores likely record thousands of years of deposition (see radiocarbon dating of North Stromatolite Lake and other nearby systems [Raudsepp et al. 2022] and a discussion of regional carbonate lake sedimentation rates [Skinner et al. 1963]). Thus, our pH measurements, which were taken during the heavy rainfall in the austral winter, are unlikely to represent the time‐averaged chemistry of the paleolakes as reflected in our bulk sediment chemistry. We recommend that future work tracks fundamental water chemistry parameters (including pH, salinity, and temperature) of designated lakes on a semi‐monthly basis. Beyond improving the resolving power of our data, this addition could help in assessing (1) the impacts of anthropogenic climate change on the systems (by comparing them to existing geochemical records) and (2) whether the extent of pH and/or salinity fluctuations is directly tied to the predominant microbial ecologies and macroscopic fauna and flora distributions at each site.

The accumulation of ammonium in the water column may play a larger role for δ^15^N elevation than does absolute pH at these sites. For example, at a pH of 8.5 and a temperature of 30°C, an equilibrated, ideal NH_4_^+^–NH_3_ solution would be 20.3% un‐ionized NH_3_ (Thurston et al. 1979). Equation (1), which is based on experimental results from a previous study (Li et al. 2012), indicates that the fractionation under those conditions would be 1000lnα ≈ 43.3.

(1) $$\begin{array}{c} 1000\text{ln}\alpha_{{\text{NH}}_{4}^{+}–{\mathrm{NH}}_{3(\mathrm{aq})}}=25.94\times \left(10^{3}/T\right)-42.25 \end{array}$$

In that same study, the effect was observed to follow batch equilibrium fractionation (Equation (2); as opposed to kinetic):

(2) $$\begin{array}{c} 1000\text{ln}\alpha_{{\text{NH}}_{4}^{+}–{\mathrm{NH}}_{3(\mathrm{aq})}}=\left(\delta^{15}N_{\text{final}}–\delta^{15}N_{\text{initial}}\right)/\left(1-f\right) \end{array}$$

where δ^15^N_initial_ and δ^15^N_final_ are the isotopic compositions of the initial and remaining ammonium, respectively, and *f* refers to the molar fraction of remaining ammonium after a portion is converted to ammonia. Therefore, if we conservatively assume an initial δ^15^N_ammonium_ = 5‰ (the average δ^15^N value of marine nitrate), then the volatilization of 20.3% of NH_*x*_ from solution would result in residual NH_4_^+^ with a δ^15^N = 13.8‰ at the pH of 8.5. This isotope value is in good agreement with previous interpretations of ammonia volatilization in both modern and ancient systems, despite pH being below those typically invoked (pH ≥ 9.25) for such an effect. Thus the use of pH ≥ 9.25 is an oversimplification, as pH modulates the extent of fractionation rather than setting a threshold for the volatilization effect. However, a sufficiently large reservoir of isotopically heavy NH_4_^+^ is needed to preserve the heightened δ^15^N value in sediments. Specifically, the reservoir needs to be large enough to feed a significant fraction of the biological community (Knapp 2012). If the absolute amount of dissolved ammonium is less than 2–10 μM (Darnajoux et al. 2022), N_2_ fixation is favored, which generates biomass with a δ^15^N value near zero permil and would thus dilute the isotopic signature of ammonium‐assimilators within sediments. This mechanism also explains why many aqueous environments, including the ocean (pH ≈ 8.1), have pH levels that should impart some degrees of ammonia generation but do not express it in terms of δ^15^N. If circumstances do not favor the accumulation of ammonium and/or allow for unidirectional escape of ammonia from a closed‐basin setting, then it is unlikely that volatilization will manifest in the δ^15^N record.

Therefore, evidence of ammonia volatilization in the sediments of shallow, oxic lakes may indicate that the system: (1) was basic (e.g., pH ≥ 8.5, as in the above example), (2) was hypersaline to an extent that it significantly diminished nitrification even under oxic conditions (i.e., ≥ 100 PSU), and (3) had ammonium contents that were high enough (> 2–10 μM) to avoid dilution of the isotopic signal by nitrogen fixation. Future work should focus on incorporating other important parameters for ammonia escape in natural systems, including microbial ammonia uptake via diffusion (Ritchie 2013), relative humidity, and wind perturbation. One eventual utility of δ^15^N in the sediments of such lakes could be to constrain paleo pH, whereby uncertainty is based in part on water‐column redox and the plausible temperature range (which affects the output value of Equation (1)).

### 5.6 The Astrobiological Value of Nitrogen Cycling in Shallow, Alkaline‐Hypersaline Lakes

Life may have originated in part through the abiotic development of large organic polymers from small molecular building blocks as facilitated by repeated wet‐dry cycles in a shallow aqueous environment, and the evapo‐concentration of critical biomolecules is a common aspect of proposed origin‐of‐life mechanisms (Damer and Deamer 2020; Frenkel‐Pinter et al. 2021; Menor Salván et al. 2020; Toner and Catling 2019, 2020). The combination of abiotic polymerization reactions of organic carbon molecules and the self‐assembly of membranous compartments appears to occur more readily in acidic environments (Deamer et al. 2019). However, alkaline systems are the more favorable setting for the accumulation of phosphate, an essential but relatively scarce molecule needed by all life (Toner and Catling 2020).

The δ^15^N record of ancient closed‐basin lakes may help identify hypersaline and high pH conditions and thereby distinguish those settings from hypersaline and alkaline locations lacking very high pH. The combined focus on alkalinity and pH as discrete parameters is important to discussions about early Earth or Mars > 3.5Ga when surface warmth on both planets was likely supported by the insulation of a thick CO_2_ atmosphere (Catling and Zahnle 2020; Wordsworth et al. 2021). Equilibration of high atmospheric *p*CO_2_ with surface waters can drive pH down (pH < 7; Halevy and Bachan 2017; Krissansen‐Totton et al. 2018) while at the same time contributing to elevated carbonate alkalinity through weathering processes (Toner and Catling 2020; Hurowitz et al. 2023). Thus, independent proxies for pH and alkalinity could fingerprint high atmospheric CO_2_, for example on early Mars (Stüeken et al. 2020).

Further, our results at the Coorong lakes show that elevated δ^15^N in ancient settings cannot be used as direct evidence for anoxic bottom waters (i.e., denitrification). Separate lines of evidence are needed, such as iron‐based paleoredox proxies (Raiswell et al. 2018). The mechanism described in Section 5.5 applies to hypersaline systems lacking redox stratification and thus the effects of denitrification on δ^15^N are minimal. The capacity for denitrification to contribute to elevated δ^15^N must always be evaluated because ammonia volatilization can also occur in redox‐stratified settings. Examples include Holocene Lonar and Devils lakes (located in central India and North Dakota, USA, respectively; Lent et al. 1995; Menzel et al. 2013) and the ancient Miocene Ries crater lake and Permian Fengcheng formation (Stüeken et al. 2020; Xia et al. 2020). In each of those studies, independent data point to at least transiently anoxic conditions in the water column, and so the potential role of denitrification must be addressed (e.g., Stüeken et al. 2020). Conversely, the Coorong lakes demonstrate that naturally alkaline‐hypersaline (but otherwise oxic) water columns can produce and preserve high δ^15^N values in the sedimentary record.

## 6 Conclusion

An investigation of the sedimentary δ^15^N of shallow, alkaline lakes in the Coorong region has uncovered a significant amount of intersystem isotopic variation. These differences include δ^15^N values that extend up to 18.6‰, which are substantially higher than data from the vast majority of both modern and ancient sediments. Contextual data, primarily in the form of major element abundances and other stable isotope ratios (δ^13^C_carb_, δ^18^O_carb_, and δ^13^C_org_), provide a means of interpreting the key biogeochemical parameters that drive the intersystem δ^15^N variation. The most significant correlations involve salinity‐associated elements (SAEs), particularly Na and Sr. We posit that this relationship is linked to the well‐studied effects of Na‐induced dampening of microbial nitrification rates, favoring the accumulation of ammonic nitrogen. Principal component analyses suggest that high Sr may hinder nitrogen cycling. However, if real, the effects of Sr toxicity on nitrifying bacteria are unknown and warrant further study. Closed‐basin behavior, as interpreted via δ^13^C_carb_–δ^18^O_carb_ correlations, was inconsistent across the study sites. This is true despite the observation that most of the systems have no apparent outlet. However, Halite Lake, which consistently had the highest δ^15^N of any site, displayed a strong positive relationship (*R* ^2^ = 0.78, *p* < 0.001) between δ^13^C_carb_ and δ^18^O_carb_. Halite Lake also appears to be evapo‐concentrated from seawater, in agreement with previous sedimentological and mineralogical evidence (Warren 1990). Ultimately, we have proposed a novel mechanism for δ^15^N elevation (Figure 7) that incorporates findings from previous work done on salterns (Isaji et al. 2019) but adds the likely relevance of cation substitution in clays and the degradation of organic matter near the sediment–water interface during early diagenesis.

Evaluating ammonia volatilization via δ^15^N can be a powerful indicator of high pH, particularly if contextual evidence in these systems also supports closed‐basin behavior and hypersalinity. While not a strict prerequisite, hypersalinity may enhance elevated δ^15^N signals and their preservation, as well as extend their relevance into settings with oxic water columns. This is due to the ammonium replete and NO_*x*_^−^ scarce conditions in many hypersaline waters, a phenomenon that has been observed for several decades and occasionally linked to the inhibition of microbial nitrification. Such constraints on pH, salinity, and basin restriction, particularly when used in combination, may inform not only nutrient cycling but also paleoatmospheric *p*CO_2_, as well as the possible conditions associated with prebiotic milieus that have been proposed to have facilitated the origin of life in alkaline settings. Our study exemplifies how leveraging well‐studied modern environments as calibration sites can provide a much‐needed expansion of interpretive geochemical frameworks.

## Conflicts of Interest

The authors declare no conflicts of interest.

## Supporting information


Data S1.

## Data Availability

All data reported in this manuscript are included in Supplemental Tables S1–S4. All data needed to evaluate the conclusions in the paper are present in the paper and Suporting Information. Additional data or information related to this paper may be requested from the authors.
