# Supplementary material for: Elevated δ15N Linked to Inhibited Nitrification Coupled to Ammonia Volatilization in Sediments of Shallow Alkaline‐Hypersaline Lakes
Source: Geobiology. 2025 Apr 4;23(2):e70018. doi: 10.1111/gbi.70018 (PMC11970551; doi:10.1111/gbi.70018)

Supplementary material for “Elevated δ^15^N linked to inhibited nitrification coupled to ammonia volatilization in sediments of shallow alkaline-hypersaline lakes”

**Table S1** Stable isotope ratio values (δ^15^N, δ^13^C_org_, δ^13^C_carb_, and δ^18^O_carb_) from all sample sites. Reproducibilities (1SD) are: ± 0.5‰ or better for δ^15^N and δ^13^C_org_, except for sample “NL-8” which showed ± 1.2‰; ± 0.1‰ or better for δ^13^C_carb_ and ± 0.2‰ or better for δ^18^O_carb_.

| **Identifier** | **δ^15^N (‰)** | **δ^13^C_org_ (‰)** | **δ^13^C_carb_ (‰)** | **δ^18^O_carb_ (‰)** | **Depth (cm)** |
| --- | --- | --- | --- | --- | --- |
| *Cantara South Lake:* | |  |  |  |  |
| CS1 | 6.9 | -16.4 | -0.3 | 33.6 | 0 – 2 |
| CS2 | 7.2 | -17.7 | 0.5 | 34.5 | 2 – 4 |
| CS3 | 7.5 | -18.3 | 0.7 | 34.9 | 4 – 6 |
| CS4 | 8.0 | -18.9 | 0.7 | 35.4 | 6 – 8 |
| CS5 | 8.1 | -19.1 | 0.7 | 35.3 | 8 – 10 |
| CS6 | 10.1 | -19.5 | 0.0 | 35.8 | 10 – 12 |
| CS7 | 9.2 | -19.1 | 0.1 | 35.6 | 12 – 14 |
| CS8 | 9.2 | -18.7 | 0.0 | 35.1 | 14 – 16 |
| CS9 | 9.6 | -18.4 | 0.2 | 35.2 | 16 – 18 |
| CS10 | 9.0 | -17.6 | 0.0 | 35.3 | 18 – 20 |
| CS11 | 8.0 | -17.4 | 0.2 | 34.8 | 20 – 22 |
| CS12 | 5.9 | -16.7 | 0.4 | 35.3 | 22 – 24 |
| CS13 | 7.3 | -17.5 | 0.3 | 34.9 | 24 – 26 |
|  |  |  |  |  |  |
| *Dolomite Lake:* | | |  |  |  |
| DL1 | 7.9 | -22.6 | -2.6 | 36.9 | 0 – 2 |
| DL2 | 8.7 | -22.8 | -2.9 | 37.3 | 2 – 4 |
| DL3 | 9.4 | -22.5 | -3.2 | 36.3 | 4 – 6 |
| DL4 | 8.7 | -21.8 | -2.9 | 36.0 | 6 – 8 |
| DL5 | 8.6 | -21.6 | -2.1 | 36.0 | 8 – 10 |
| DL6 | 9.1 | -20.8 | -1.7 | 35.9 | 10 – 12 |
| DL7 | 8.7 | -20.7 | -1.5 | 35.9 | 12 – 14 |
| DL8 | 8.3 | -20.9 | -1.2 | 36.4 | 14 – 16 |
| DL9 | 8.9 | -20.7 | -1.2 | 35.8 | 16 – 18 |
| DL10 | 6.7 | -21.0 | -0.8 | 36.0 | 18 – 20 |
| DL11 | 8.4 | -21.0 | -0.8 | 35.8 | 20 – 22 |
| DL12 | 7.3 | -21.3 | -0.6 | 36.5 | 22 – 24 |
| DL13 | 5.7 | -20.5 | 0.7 | 35.6 | 24 – 26 |
| DL14 | 4.0 | -20.7 | 1.1 | 35.6 | 26 – 28 |
| DL15 | 5.6 | -21.8 | 0.3 | 35.7 | 28 – 30 |
| DL16 | 3.8 | -20.9 | 0.6 | 36.1 | 30 – 32 |
|  |  |  |  |  |  |
| *Halite Lake:* | |  |  |  |  |
| HL1 | 10.7 | -20.3 | 3.1 | 34.8 | 0 – 2 |
| HL2 | 12.9 | -20.9 | 3.6 | 34.7 | 2 – 4 |
| HL3 | 12.8 | -19.5 | 4.1 | 35.1 | 4 – 6 |
| HL4 | 13.1 | -19.6 | 5.0 | 35.0 | 6 – 8 |
| HL5 | 13.2 | -19.6 | 4.8 | 35.6 | 8 – 10 |
| HL6 | 14.7 | -19.6 | 4.5 | 34.6 | 10 – 12 |
| HL7 | 17.9 | -20.7 | 4.2 | 35.0 | 12 – 14 |
| HL8 | 17.6 | -20.3 | 4.5 | 34.3 | 14 – 16 |
| HL9 | 17.8 | -19.0 | 3.7 | 34.4 | 16 – 18 |
| HL10 | 17.7 | -19.4 | 3.4 | 33.8 | 18 – 20 |
| HL11 | 17.9 | -19.3 | 3.1 | 33.7 | 20 – 22 |
| HL12 | 16.8 | -20.0 | 2.4 | 33.4 | 22 – 24 |
| HL13 | 18.3 | -20.4 | 1.7 | 33.5 | 24 – 26 |
| HL14 | 17.6 | -20.3 | 1.5 | 33.2 | 26 – 28 |
| HL15 | 18.6 | -21.0 | 0.7 | 33.4 | 28 – 30 |
|  |  |  |  |  |  |
| *Mini Dolomite Lake:* | | |  |  |  |
| MD1 | 8.6 | -20.7 | 1.3 | 36.1 | 0 – 2 |
| MD2 | 9.3 | -21.2 | 1.3 | 37.0 | 2 – 4 |
| MD3 | 10.2 | -21.8 | 1.4 | 36.6 | 4 – 6 |
| MD4 | 10.6 | -22.1 | 0.6 | 35.8 | 6 – 8 |
| MD5 | 10.0 | -21.1 | 0.2 | 35.3 | 8 – 10 |
| MD6 | 10.4 | -20.7 | -0.1 | 34.6 | 10 – 12 |
| MD7 | 10.9 | -20.7 | 0.1 | 34.6 | 12 – 14 |
| MD8 | 10.6 | -20.0 | 0.4 | 34.6 | 14 – 16 |
| MD9 | 10.4 | -20.5 | 0.7 | 35.7 | 16 – 18 |
| MD10 | 10.1 | -19.7 | 1.2 | 35.5 | 18 – 20 |
|  |  |  |  |  |  |
| *North Stromatolite Lake:* | | |  |  |  |
| NS1 | 9.0 | -22.4 | 1.4 | 34.7 | 0 – 2 |
| NS2 | 9.6 | -22.8 | 1.8 | 35.6 | 2 – 4 |
| NS3 | 11.6 | -24.8 | 1.7 | 35.3 | 4 – 6 |
| NS4 | 11.9 | -25.7 | 1.5 | 36.0 | 6 – 8 |
| NS5 | 12.0 | -24.4 | 1.3 | 34.8 | 8 – 10 |
| NS6 | 12.9 | -24.7 | 1.5 | 34.2 | 10 – 12 |
| NS7 | 13.7 | -23.3 | 1.5 | 34.1 | 12 – 14 |
| NS8 | 14.0 | -22.7 | 1.7 | 35.4 | 14 – 16 |
| NS9 | 12.8 | -23.4 | 2.0 | 35.0 | 16 – 18 |
| NS10 | 13.4 | -23.5 | 1.7 | 35.4 | 18 – 20 |
| NS11 | 13.7 | -22.5 | 2.7 | 35.6 | 20 – 22 |
| NS13 | 11.7 | -22.0 | 2.1 | 35.2 | 24 – 26 |
| NS14 | 10.4 | -23.3 | 1.3 | 37.0 | 26 – 28 |
| NS15 | 9.5 | -21.7 | 1.1 | 36.0 | 28 – 30 |
|  |  |  |  |  |  |
| *North Lagoon:* | | |  |  |  |
| NL1 | 7.5 | -25.3 | -0.3 | 32.2 | 0 – 2 |
| NL2 | 6.9 | -25.4 | -1.0 | 32.5 | 2 – 4 |
| NL3 | 7.4 | -24.5 | -1.0 | 32.7 | 4 – 6 |
| NL4 | 6.2 | -25.2 | -1.0 | 31.7 | 6 – 8 |
| NL5 | 6.9 | -25.2 | -0.5 | 32.0 | 8 – 10 |
| NL6 | 8.2 | -25.1 | -1.1 | 30.6 | 10 – 12 |
| NL7 | 7.6 | -25.1 | 0.0 | 31.9 | 12 – 14 |
| NL8 | 7.4 | -25.7 | 0.8 | 31.8 | 14 – 16 |
| NL9 | 6.5 | -24.6 | 0.8 | 32.1 | 16 – 18 |

**Table S2** Nitrogen and carbon contents (TN_decarb_, TIC, TOC, and C/N) of all sample sites in this study. Reproducibilities (1SD) are: ± 0.06% or better for TN_decarb_, better than ± 0.1% for both TIC and TOC. C/N (mol/mol) is derived directly from measurement of decarbonated materials combusted in an elemental analyzer prior to isotope ratio measurement; it is not the ratio of TOC to TN_decarb_.

| **Identifier** | **TN_decarb_ (wt%)** | **TIC (wt%)** | **TOC (wt%)** | **C/N (mol/mol)** | **Depth (cm)** | |  |  |  |  |  |
| --- | --- | --- | --- | --- | --- | --- | --- | --- | --- | --- | --- |
| *Cantara South Lake:* | |  |  |  |  | |  |  |  |  |  |
| CS1 | 1.18 | 6.4 | 7.3 | 24.6 | 0 – 2 | |  |  |  |  |  |
| CS2 | 0.14 | 7.7 | 1.1 | 16.4 | 2 – 4 | |  |  |  |  |  |
| CS3 | 0.09 | 8.4 | 0.8 | 16.0 | 4 – 6 | |  |  |  |  |  |
| CS4 | 0.05 | 8.9 | 0.3 | 15.0 | 6 – 8 | |  |  |  |  |  |
| CS5 | 0.03 | 9.0 | 0.4 | 15.0 | 8 – 10 | |  |  |  |  |  |
| CS6 | 0.18 | 9.3 | 1.0 | 13.6 | 10 – 12 | |  |  |  |  |  |
| CS7 | 0.35 | 9.6 | 0.7 | 14.3 | 12 – 14 | |  |  |  |  |  |
| CS8 | 0.33 | 9.5 | 0.9 | 14.9 | 14 – 16 | |  |  |  |  |  |
| CS9 | 0.24 | 9.4 | 0.7 | 16.7 | 16 – 18 | |  |  |  |  |  |
| CS10 | 0.32 | 8.9 | 1.1 | 17.4 | 18 – 20 | |  |  |  |  |  |
| CS11 | 0.49 | 9.0 | 1.3 | 19.0 | 20 – 22 | |  |  |  |  |  |
| CS12 | 1.10 | 8.5 | 2.8 | 17.2 | 22 – 24 | |  |  |  |  |  |
| CS13 | 0.65 | 8.8 | 1.5 | 18.5 | 24 – 26 | |  |  |  |  |  |
|  |  |  |  |  |  | |  |  |  |  |  |
| *Dolomite Lake:* | |  |  |  |  | |  |  |  |  |  |
| DL1 | 0.07 | 10.3 | 0.5 | 12.7 | 0 – 2 | |  |  |  |  |  |
| DL2 | 0.05 | 10.4 | 0.3 | 13.1 | 2 – 4 | |  |  |  |  |  |
| DL3 | 0.04 | 10.2 | 0.1 | 14.5 | 4 – 6 | |  |  |  |  |  |
| DL4 | 0.06 | 9.2 | 0.8 | 14.7 | 6 – 8 | |  |  |  |  |  |
| DL5 | 0.04 | 8.9 | 0.5 | 16.0 | 8 – 10 | |  |  |  |  |  |
| DL6 | 0.06 | 8.3 | 0.5 | 16.5 | 10 – 12 | |  |  |  |  |  |
| DL7 | 0.03 | 8.7 | 0.2 | 16.5 | 12 – 14 | |  |  |  |  |  |
| DL8 | 0.01 | 8.3 | 0.4 | 20.0 | 14 – 16 | |  |  |  |  |  |
| DL9 | 0.01 | 8.3 | 0.1 | 20.4 | 16 – 18 | |  |  |  |  |  |
| DL10 | 0.02 | 8.5 | 0.0 | 18.3 | 18 – 20 | |  |  |  |  |  |
| DL11 | 0.02 | 9.3 | 0.0 | 19.0 | 20 – 22 | |  |  |  |  |  |
| DL12 | 0.02 | 9.1 | 0.0 | 20.4 | 22 – 24 | |  |  |  |  |  |
| DL13 | 0.01 | 6.1 | 0.2 | 19.6 | 24 – 26 | |  |  |  |  |  |
| DL14 | 0.02 | 4.3 | 0.3 | 23.6 | 26 – 28 | |  |  |  |  |  |
| DL15 | 0.01 | 5.4 | 0.2 | 22.0 | 28 – 30 | |  |  |  |  |  |
| DL16 | 0.01 | 2.1 | 0.0 | 23.6 | 30 – 32 | |  |  |  |  |  |
|  |  |  |  |  |  | |  |  |  |  |  |
| *Halite Lake:* | |  |  |  |  | |  |  |  |  |  |
| HL1 | 0.15 | 6.5 | 1.5 | 10.3 | 0 – 2 | |  |  |  |  |  |
| HL2 | 0.08 | 6.1 | 0.9 | 12.4 | 2 – 4 | |  |  |  |  |  |
| HL3 | 0.28 | 8.6 | 0.8 | 11.1 | 4 – 6 | |  |  |  |  |  |
| HL4 | 0.40 | 8.0 | 0.8 | 11.9 | 6 – 8 | |  |  |  |  |  |
| HL5 | 0.64 | 8.0 | 1.0 | 11.2 | 8 – 10 | |  |  |  |  |  |
| HL6 | 0.71 | 7.5 | 1.3 | 11.6 | 10 – 12 | |  |  |  |  |  |
| HL7 | 0.24 | 8.0 | 0.9 | 13.4 | 12 – 14 | |  |  |  |  |  |
| HL8 | 0.59 | 8.2 | 1.0 | 13.2 | 14 – 16 | |  |  |  |  |  |
| HL9 | 1.67 | 8.5 | 1.2 | 12.9 | 16 – 18 | |  |  |  |  |  |
| HL10 | 0.33 | 8.7 | 1.3 | 13.5 | 18 – 20 | |  |  |  |  |  |
| HL11 | 0.48 | 8.7 | 1.1 | 13.2 | 20 – 22 | |  |  |  |  |  |
| HL12 | 0.84 | 8.7 | 0.9 | 15.8 | 22 – 24 | |  |  |  |  |  |
| HL13 | 0.56 | 8.2 | 1.5 | 16.9 | 24 – 26 | |  |  |  |  |  |
| HL14 | 0.74 | 7.6 | 2.1 | 18.3 | 26 – 28 | |  |  |  |  |  |
| HL15 | 0.71 | 7.8 | 1.1 | 18.8 | 28 – 30 | |  |  |  |  |  |
|  |  |  |  |  |  | |  |  |  |  |  |
| *Mini Dolomite Lake:* | |  |  |  |  | |  |  |  |  |  |
| MD1 | 0.04 | 8.7 | 1.0 | 17.7 | 0 – 2 | |  |  |  |  |  |
| MD2 | 0.15 | 10.1 | 1.0 | 18.3 | 2 – 4 | |  |  |  |  |  |
| MD3 | 0.08 | 9.5 | 1.1 | 16.2 | 4 – 6 | |  |  |  |  |  |
| MD4 | 0.06 | 9.0 | 0.6 | 13.8 | 6 – 8 | |  |  |  |  |  |
| MD5 | 0.15 | 9.1 | 1.0 | 13.8 | 8 – 10 | |  |  |  |  |  |
| MD6 | 0.15 | 9.0 | 0.7 | 15.6 | 10 – 12 | |  |  |  |  |  |
| MD7 | 0.14 | 8.8 | 0.7 | 15.7 | 12 – 14 | |  |  |  |  |  |
| MD8 | 0.14 | 8.4 | 1.1 | 16.4 | 14 – 16 | |  |  |  |  |  |
| MD9 | 0.11 | 9.4 | 0.6 | 16.3 | 16 – 18 | |  |  |  |  |  |
| MD10 | 0.05 | 9.8 | 0.4 | 18.3 | 18 – 20 | |  |  |  |  |  |
|  |  |  |  |  |  | |  |  |  |  |  |
| *North Stromatolite Lake:* | |  |  |  |  | |  |  |  |  |  |
| NS1 | 0.06 | 8.4 | 0.3 | 13.1 | 0 – 2 | |  |  |  |  |  |
| NS2 | 0.04 | 8.4 | 0.4 | 16.4 | 2 – 4 | |  |  |  |  |  |
| NS3 | 0.04 | 8.1 | 0.4 | 17.4 | 4 – 6 | |  |  |  |  |  |
| NS4 | 0.05 | 8.0 | 0.4 | 19.8 | 6 – 8 | |  |  |  |  |  |
| NS5 | 0.03 | 7.6 | 0.2 | 18.3 | 8 – 10 | |  |  |  |  |  |
| NS6 | 0.03 | 7.9 | 0.4 | 20.9 | 10 – 12 | |  |  |  |  |  |
| NS7 | 0.04 | 8.5 | 0.3 | 19.8 | 12 – 14 | |  |  |  |  |  |
| NS8 | 0.03 | 8.4 | 0.2 | 19.4 | 14 – 16 | |  |  |  |  |  |
| NS9 | 0.06 | 8.8 | 0.3 | 19.3 | 16 – 18 | |  |  |  |  |  |
| NS10 | 0.04 | 8.9 | 0.1 | 19.8 | 18 – 20 | |  |  |  |  |  |
| NS11 | 0.06 | 9.5 | 0.2 | 20.3 | 20 – 22 | |  |  |  |  |  |
| NS13 | 0.02 | 8.5 | 0.1 | 21.9 | 24 – 26 | |  |  |  |  |  |
| NS14 | 0.03 | 8.1 | 0.5 | 23.0 | 26 – 28 | |  |  |  |  |  |
| NS15 | 0.05 | 7.7 | 0.7 | 21.3 | 28 – 30 | |  |  |  |  |  |
|  |  |  |  |  |  |  | |  |  |  |  |
| *North Lagoon:* | |  |  |  |  |  | |  |  |  |  |
| NL1 | 0.16 | 2.4 | 2.1 | 14.0 | 0 – 2 | |  |  |  |  |  |
| NL2 | 0.22 | 3.4 | 2.1 | 12.9 | 2 – 4 | |  |  |  |  |  |
| NL3 | 0.03 | 3.4 | 0.7 | 14.0 | 4 – 6 | |  |  |  |  |  |
| NL4 | 0.02 | 3.0 | 0.5 | 15.9 | 6 – 8 | |  |  |  |  |  |
| NL5 | 0.01 | 2.3 | 0.4 | 17.5 | 8 – 10 | |  |  |  |  |  |
| NL6 | 0.02 | 2.9 | 0.3 | 16.0 | 10 – 12 | |  |  |  |  |  |
| NL7 | 0.01 | 2.4 | 0.2 | 18.0 | 12 – 14 | |  |  |  |  |  |
| NL8 | 0.01 | 1.8 | 0.2 | 16.4 | 14 – 16 | |  |  |  |  |  |
| NL9 | 0.01 | 2.2 | 0.1 | 15.8 | 16 – 18 | |  |  |  |  |  |

**Table S3** All reported ICP-MS major element abundances (Na, Mg, K, Ca, and Sr) in this study. Reproducibilities (1SD) are: ± 0.26% or better for Na; ± 0.86% or better for Mg; ± 0.32% or better for K; ± 1.04% or better for Ca; and ± 0.07% or better for Sr.

| **Identifier** | **Na**  **(wt%)** | **Mg (wt%)** | **K (wt%)** | **Ca (wt%)** | **Sr (wt%)** | **Depth (cm)** |
| --- | --- | --- | --- | --- | --- | --- |
| *Cantara South Lake:* | |  |  |  |  |  |
| CS1 | 5.79 | 4.52 | 0.55 | 11.62 | 0.40 | 0 – 2 |
| CS2 | 1.05 | 2.66 | 0.23 | 10.77 | 0.24 | 2 – 4 |
| CS3 | 2.10 | 6.37 | 0.39 | 18.78 | 0.66 | 4 – 6 |
| CS4 | 3.60 | 10.82 | 0.51 | 28.25 | 1.06 | 6 – 8 |
| CS5 | 1.78 | 5.73 | 0.20 | 13.95 | 0.70 | 8 – 10 |
| CS6 | 3.20 | 7.02 | 0.21 | 14.14 | 0.80 | 10 – 12 |
| CS7 | 3.87 | 7.32 | 0.34 | 14.97 | 0.80 | 12 – 14 |
| CS8 | 3.81 | 6.51 | 0.36 | 15.55 | 0.54 | 14 – 16 |
| CS9 | 3.86 | 6.69 | 0.16 | 15.48 | 0.47 | 16 – 18 |
| CS10 | 4.40 | 6.29 | 0.17 | 15.44 | 0.79 | 18 – 20 |
| CS11 | 4.81 | 6.65 | 0.17 | 16.21 | 0.42 | 20 – 22 |
| CS12 | 5.52 | 6.36 | 0.28 | 16.08 | 0.46 | 22 – 24 |
| CS13 | 5.23 | 7.18 | 0.50 | 16.01 | 0.40 | 24 – 26 |
|  |  |  |  |  |  |  |
| *Dolomite Lake:* | | |  |  |  |  |
| DL1 | 0.91 | 9.36 | 0.24 | 11.30 | 0.22 | 0 – 2 |
| DL2 | 1.45 | 9.35 | 0.27 | 11.53 | 0.18 | 2 – 4 |
| DL3 | 1.82 | 9.14 | 0.05 | 11.67 | 0.20 | 4 – 6 |
| DL4 | 2.53 | 6.66 | 0.23 | 12.01 | 0.23 | 6 – 8 |
| DL5 | 2.05 | 5.24 | 0.11 | 12.06 | 0.26 | 8 – 10 |
| DL6 | 1.67 | 4.21 | 0.08 | 12.69 | 0.28 | 10 – 12 |
| DL7 | 1.76 | 3.49 | 0.01 | 13.47 | 0.25 | 12 – 14 |
| DL8 | 1.57 | 3.17 | 0.13 | 13.96 | 0.26 | 14 – 16 |
| DL9 | 1.38 | 2.69 | 0.13 | 12.98 | 0.25 | 16 – 18 |
| DL10 | 1.65 | 2.97 | 0.07 | 17.03 | 0.26 | 18 – 20 |
| DL11 | 1.95 | 3.83 | 0.00 | 17.60 | 0.27 | 20 – 22 |
| DL12 | 1.72 | 3.19 | 0.08 | 18.21 | 0.26 | 22 – 24 |
| DL13 | 1.93 | 1.33 | 0.15 | 14.62 | 0.27 | 24 – 26 |
| DL14 | 1.91 | 0.92 | 0.17 | 10.39 | 0.25 | 26 – 28 |
| DL15 | 1.80 | 1.28 | 0.16 | 13.64 | 0.18 | 28 – 30 |
| DL16 | 1.12 | 0.46 | 0.14 | 4.82 | 0.09 | 30 – 32 |
|  |  |  |  |  |  |  |
| *Halite Lake:* | |  |  |  |  |  |
| HL1 | 8.99 | 4.83 | 0.26 | 10.26 | 1.32 | 0 – 2 |
| HL2 | 4.94 | 6.48 | 0.00 | 7.37 | 0.97 | 2 – 4 |
| HL3 | 5.09 | 9.77 | 0.00 | 9.96 | 1.49 | 4 – 6 |
| HL4 | 6.63 | 11.27 | 0.19 | 7.24 | 1.19 | 6 – 8 |
| HL5 | 6.84 | 11.95 | 0.12 | 7.98 | 1.22 | 8 – 10 |
| HL6 | 9.27 | 11.03 | 0.53 | 9.16 | 1.49 | 10 – 12 |
| HL7 | 9.56 | 13.56 | 0.65 | 10.89 | 1.53 | 12 – 14 |
| HL8 | 8.43 | 12.11 | 0.73 | 10.48 | 1.47 | 14 – 16 |
| HL9 | 9.57 | 7.96 | 0.97 | 16.79 | 2.14 | 16 – 18 |
| HL10 | 7.38 | 3.16 | 0.96 | 16.20 | 1.68 | 18 – 20 |
| HL11 | 6.83 | 2.28 | 0.84 | 19.50 | 1.75 | 20 – 22 |
| HL12 | 7.59 | 2.34 | 0.74 | 15.27 | 1.32 | 22 – 24 |
| HL13 | 6.33 | 1.83 | 0.66 | 13.62 | 1.10 | 24 – 26 |
| HL14 | 6.26 | 2.35 | 0.95 | 15.17 | 1.68 | 26 – 28 |
| HL15 | 7.27 | 2.24 | 0.81 | 13.49 | 1.65 | 28 – 30 |
|  |  |  |  |  |  |  |
| *Mini Dolomite Lake:* | |  |  |  |  |  |
| MD1 | 1.07 | 8.52 | 0.24 | 10.96 | 0.64 | 0 – 2 |
| MD2 | 1.63 | 11.40 | 0.28 | 11.94 | 0.80 | 2 – 4 |
| MD3 | 2.09 | 11.36 | 0.42 | 12.62 | 0.76 | 4 – 6 |
| MD4 | 2.18 | 9.29 | 0.22 | 12.11 | 0.57 | 6 – 8 |
| MD5 | 2.66 | 9.32 | 0.23 | 12.69 | 0.45 | 8 – 10 |
| MD6 | 2.70 | 7.61 | 0.23 | 11.72 | 0.38 | 10 – 12 |
| MD7 | 3.78 | 9.55 | 0.52 | 14.72 | 0.49 | 12 – 14 |
| MD8 | 3.29 | 7.61 | 0.31 | 13.03 | 0.44 | 14 – 16 |
| MD9 | 2.60 | 6.85 | 0.28 | 15.10 | 0.56 | 16 – 18 |
| MD10 | 2.26 | 4.84 | 0.34 | 17.36 | 0.63 | 18 – 20 |
|  |  |  |  |  |  |  |
| *North Stromatolite Lake:* | |  |  |  |  |  |
| NS1 | 1.55 | 5.76 | 0.16 | 16.25 | 1.79 | 0 – 2 |
| NS2 | 3.91 | 7.20 | 0.22 | 23.01 | 2.35 | 2 – 4 |
| NS3 | 3.66 | 7.67 | 0.24 | 21.06 | 2.25 | 4 – 6 |
| NS4 | 3.39 | 6.94 | 0.26 | 20.69 | 2.16 | 6 – 8 |
| NS5 | 4.22 | 8.83 | 0.13 | 21.68 | 2.25 | 8 – 10 |
| NS6 | 2.43 | 7.69 | 0.19 | 7.80 | 0.60 | 10 – 12 |
| NS7 | 3.99 | 11.28 | 0.16 | 12.49 | 0.92 | 12 – 14 |
| NS8 | 3.00 | 5.68 | 0.31 | 9.96 | 0.88 | 14 – 16 |
| NS9 | 3.98 | 7.66 | 0.28 | 19.02 | 1.69 | 16 – 18 |
| NS10 | 1.40 | 5.53 | 0.11 | 13.87 | 1.59 | 18 – 20 |
| NS11 | 2.34 | 5.94 | 0.23 | 13.78 | 1.59 | 20 – 22 |
| NS13 | 1.83 | 3.02 | 0.11 | 9.46 | 1.10 | 24 – 26 |
| NS14 | 5.00 | 7.12 | 0.20 | 22.45 | 2.70 | 26 – 28 |
| NS15 | 2.49 | 4.54 | 0.15 | 13.67 | 1.61 | 28 – 30 |
|  |  |  |  |  |  |  |
| *North Lagoon:* | |  |  |  |  |  |
| NL1 | 0.85 | 0.49 | 0.69 | 3.83 | 0.08 | 0 – 2 |
| NL2 | 0.87 | 0.50 | 0.83 | 5.43 | 0.09 | 2 – 4 |
| NL3 | 0.54 | 0.38 | 0.57 | 5.23 | 0.09 | 4 – 6 |
| NL4 | 0.43 | 0.34 | 0.60 | 4.61 | 0.07 | 6 – 8 |
| NL5 | 0.30 | 0.28 | 0.80 | 3.70 | 0.05 | 8 – 10 |
| NL6 | 0.30 | 0.24 | 0.11 | 5.13 | 0.05 | 10 – 12 |
| NL7 | 0.24 | 0.14 | 0.09 | 4.41 | 0.04 | 12 – 14 |
| NL8 | 0.28 | 0.17 | 0.13 | 3.57 | 0.03 | 14 – 16 |
| NL9 | 0.33 | 0.18 | 0.08 | 5.36 | 0.05 | 16 – 18 |

**Table S4** pH values recorded during the July 2018 sampling trip of this study, along with the highest pH values measured or calculated in previous studies.


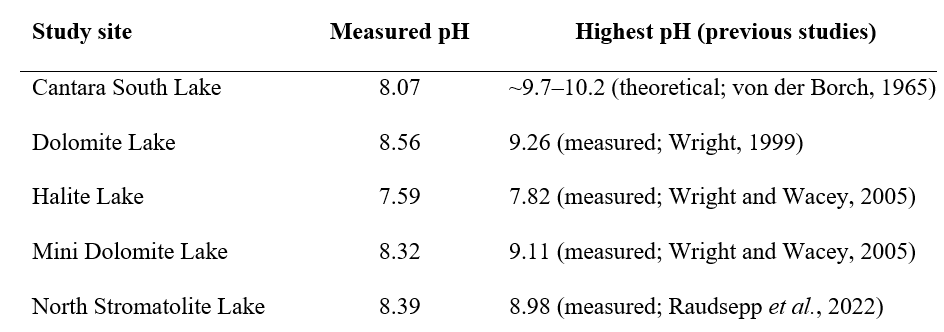

Supplement: Supplementary file 1 — Data S1. [file GBI-23-e70018-s001.zip › Tables_S1-S4.docx]
